# Designing a Health Strategy at Local Level: A Conceptual Framework for Local Governments

**DOI:** 10.3390/ijerph20136250

**Published:** 2023-06-29

**Authors:** Gonçalo Santinha, Alexandre Fernandes, Rafaela Oliveira, Nelson Pacheco Rocha

**Affiliations:** 1 GOVCOPP, Department of Social, Political and Territorial Sciences, University of Aveiro, 3810-193 Aveiro, Portugal; alexandrefernandes@ua.pt (A.F.); rafaelaoliveira@ua.pt (R.O.); 2 IEETA, Department of Medical Sciences, University of Aveiro, 3810-193 Aveiro, Portugal; npr@ua.pt

**Keywords:** decentralization, health system, healthcare governance, local government, health strategy

## Abstract

This article addresses a gap in the literature regarding the design and implementation of health and well-being strategies by local governments. It presents a conceptual framework that can help local governments to include health and well-being among their political priorities by promoting the participation of local stakeholders and the community in general. The article also highlights the important role that local governments play in public health, especially in times of crisis, such as the COVID-19 pandemic. To demonstrate the approach, the manuscript explores the recent shift toward decentralization in Portugal and the resulting emphasis on local governments leading new approaches to health governance. The planning process for Arouca’s Health Strategy, an inland municipality, is used as a case study to demonstrate the approach, which emphasizes the meaningful participation of the entire local community. The article concludes by noting that there is still significant room for improvement in all areas related to health and well-being and a need to adopt new multistakeholder governance arrangements to sustain the institutional capacity for upcoming strategies.

## 1. Introduction

Decentralization reforms have been embraced by many countries, namely in the healthcare sector, particularly since the 1980s. With the support of several international organizations, such as the United Nations and the World Bank, decentralization has been considered a way to stimulate economic growth, to improve the quality-of-service delivery, to reinforce democracy and accountability, and to increase cooperation between different sectors and stakeholders [1,2]. As decentralization implies the transfer of authority, responsibilities and services from the central government to subnational ones, governance models have been subject to change [1,3,4].

Health policies in Portugal, considered one of the most centralized countries in Europe [5], are in the hands of the Ministry of Health, which is responsible for steering the Portuguese health system but concentrates on planning, regulation, and management responsibilities [6]. Even though decentralization is referred to in the Portuguese 1976 Constitution, the few attempts to implement true reform have been unsuccessful. However, decentralization is now on the political agenda with the launch of Law n. 50/2018, demanding the transfer of power to local governments in several areas, including health. By transferring responsibilities from healthcare entities to local governments, this decentralization is initiating a process that allows these entities the possibility of including health and well-being among their political priorities by developing what the legislation calls Municipal Health Strategies. These planning strategies are important for operationalizing and delivering a local health agenda and specifically for translating national and regional goals into ground actions that can enable reductions in vulnerability and enhance communities’ quality of life. By such means, local governments can also promote the participation of local stakeholders and the community in general in planning priorities that move away from the traditional healthcare approach.

Although healthcare is not a traditional responsibility of local governments, the political response to the COVID-19 pandemic has highlighted the important role that they play in public health [7,8]. In fact, local governments are often responsible for taking the lead on many issues that affect people’s health, given their proximity to the community and responsibility for land-use and development decisions. As Crisp (2017) [9] puts it, health and healthcare are profoundly affected by other sectors and hence need to be seen in the context of education, housing, employment, environmental policies, and many other factors that help determine the health and well-being of individuals. However, in Portugal and elsewhere, local health strategic planning remains in the hands of healthcare providers, focusing more on healthcare, with other health determinants that together affect the health conditions of individuals and communities receiving significantly less attention [7,10,11].

Encompassing the transfer of new responsibilities to local governments, the process of decentralization has raised several questions on how to design health strategies at the local level and exactly what role local governments now play in the health domain, as the legislation is somewhat vague on this matter. Moreover, although the main strategic national document on health in Portugal—National Health Plan 2021–2030—emphasizes the importance of strengthening the role of municipalities in aligning territorial policies and strategies with the health sector, no specific guidelines are provided on this matter. As such, little is known about how local initiatives should be conceptualized, implemented, and evaluated [12] and, in particular, how a health strategy should be developed at the local level, considering health determinants and taking stock of their proximity to communities.

This, in turn, also implies replacing the centralized governance model with a set of constructive relationships that enable health promotion and disease prevention to be steered by local governments in cooperation with healthcare providers and other stakeholders in the same general course. Part of this role involves acknowledging the importance of empowering local government bodies to entrench a new perspective on how to improve the health conditions of individuals and communities, as well as the need for them to strengthen cooperation among different sets of local stakeholders.

This article addresses the gap in the literature regarding the design and implementation of health and well-being strategies by local governments and, accordingly, presents a conceptual framework to develop a health strategy at the local level. The implementation of such a conceptual framework is discussed for a specific local context in Portugal as an attempt to change, through practice, the dominant culture regarding the design of health policies. The results of this research also help to better pinpoint the role played by local governments with respect to healthcare regarding the improvement of communities’ quality of life.

This article is structured as follows. In the next section, the theoretical background is discussed, namely, (i) a brief conceptual approach to healthcare decentralization, naming the main advantages and disadvantages according to the literature; (ii) the description of the healthcare decentralization process in the Portuguese context; (iii) the development of a health strategy conceptual framework at the local level. Then, from an empirical point of view, the various stages that lead to the development of a health strategy at the local level are described, concluding with the presentation and discussion of the case study. Finally, the process approach, results, and limitations are discussed.

## 2. Healthcare Decentralization

The definition of decentralization is sometimes contradictory, with various authors defining it differently depending on the theoretical and practical contexts. As a malleable concept, it adapts to multiple national, regional, and local agendas [1,2,4]. 

According to Saltman et al. (2007) [4] and the OECD (2020) [6], decentralization in healthcare is the transfer of authority and decision-making power over the management, planning, and distribution and/or financing of health systems, typically from a smaller to a larger number of actors, i.e., from the central government to subnational governments. It aims to improve the overall performance of health systems and make services more responsive to citizens’ needs [1,3]. 

This concept encompasses multiple meanings and dimensions with different political implications. Regardless of the sphere in which it occurs (e.g., social, health, education), decentralization encompasses three typologies: (i) political; (ii) administrative; and (iii) fiscal. Political decentralization consists of the central government assigning some decision-making authority to subnational levels; administrative decentralization comprises the transfer of authority, operational responsibilities, and financial resources from a higher level to a lower level of the organization, but decision-making power is not fully delegated; and fiscal decentralization transfers expenditure and revenue responsibilities from central governments to subnational governments, and they are regulated by the state [6,13,14]. 

Conceptually linked and confused with decentralization, deconcentration is also a form of governance that involves the transfer of administrative responsibilities among the various levels of government. However, the decision-making authority remains at the central level, so the other levels do not acquire full autonomy [6,13]. There is nonetheless little consensus in the literature about what they actually entail and whether both concepts should be viewed in a broad and integrated way [3,4]. According to Teles (2021) [13] and the OECD (2020) [6], decentralization, in most situations, results from the combination of different typologies, be they political, administrative, or fiscal, but mostly combining all of them.

The evidence on whether decentralization achieves its goals in healthcare is mixed and ambiguous. The complexity of the concept itself and the context in which it occurs may explain the contradictory outcomes regarding its effects on equity and efficiency [3,4,14,15]. In fact, there are several factors that may have an impact on decentralization, such as the implementation process, the political system, the institutional structure, the social and cultural values, the historical-administrative context, the governance mechanisms, and the structure of the health system [3,6,13].

Research attempting to ascertain the effects of decentralization on social and economic development has tended to support the argument based on three theories [14,16,17]: (i) “voting with your feet”; (ii) “close to ground”; and (iii) “watching the watchers”. Following Charles Tiebouts’ (1956) [18] insights on using geographic movement to express one’s preferences for public goods, the first theory reflects how decentralization increases or decreases existing patterns of inequality in the distribution of people, resources, and outcomes. According to this theory, individuals choose a given location considering their personal maximization, that is, seeking an optimal cost–benefit ratio [2,19]. The second theory tracks Arrows’ (1963) [20] notion of information asymmetry in healthcare interactions and concentrates on how bringing governance closer to citizens enables the effective use of local information and community feedback for the proper and better use of resources. This proximity facilitates a greater exchange of information between the local government and citizens, achieving policies targeting real existing needs and with lower implementation costs [2]. The third theory focuses on how decentralization favors responsibilities and mutual support among multiple governance layers involving different levels of action. Encompassing the three levels of governance (constitutional, collective, and operational), this theory seeks to observe how each one interacts with the others, leaving no stakeholder out of this governance cycle [2,21]. 

The linkage between these theories and healthcare can be observed in many ways. For instance, one may have communities with more or less resources in healthcare depending on the demands of their population (“voting with your feet”). One may have governments that make decisions by considering local realities, particularly at the level of social inequalities, but depending heavily on central government funding limits their area of intervention and ability to generate or use resources (“close to ground”). One may have local governments that do not properly manage their healthcare resources, with too much spending that generates a deficit at the central government, which, in turn, also increases costs for local governments with larger budgets (“watching the watchers”) [2]. Based on these theories, it is then possible to discern a set of advantages and shortcomings of healthcare decentralization, a common basis of discussion by several authors [1,3,4,6,15].

According to the “voting with your feet” theory, the main advantages of decentralization are mostly related to improved efficiency and effectiveness in improving the quality of services, making them more oriented to citizens based on their preferences and needs. It also promotes policies that are more appropriate to local needs, also increasing the accountability of local and regional authorities for the proper management of resources. However, decentralization can also engender health inequities, since it promotes greater benefits for individuals with greater financial resources, directing services only to local elites, often through pressure groups. Moreover, decentralization can induce an increase in costs for subnational governments, which can contribute to financial deficits.

According to the “close to ground” theory, the main advantage of decentralization consists of involving the community in its own health management, making services more citizen-oriented, and adapting them to their conditions and needs. On the other hand, it contributes to greater equity in health and a higher quality of services, which, overall, will produce better health outputs and more innovation from policies. However, decentralization may raise concerns about increased costs, as policies are more focused on the individual. The introduction of new roles and responsibilities may also lead to the duplication of some services and the need for more specialized human resources, which, in turn, imply more funding from the central government to subnational levels.

Finally, according to the “watching the watchers” theory, a major advantage of decentralization is the increase in multilevel coordination between subnational governments and the central government and, from a horizontal perspective, better sectoral coordination between healthcare and other sectors. The available funding can also be shared by a larger set of actors, which, in turn, may lead to lower expenses, since everyone contributes and takes responsibility for the budget. From an adverse perspective, however, the sharing of such tasks can create shortfalls in the management of skills and responsibilities of each actor, which, in the medium and long run, may lead to less effective and efficient health services.

## 3. Health Policy Decentralization in Portugal

The Portuguese health system has been mainly based on a National Health Service (NHS) since 1979. Under the tutelage of the Ministry of Health, the NHS comprises several decentralized institutions throughout the territory, with, however, the Ministry of Health being the ultimate body for the definition and implementation of health policies. The health system in Portugal is funded primarily through general taxation, with universal coverage as a premise [22,23,24].

**Figure 1:**
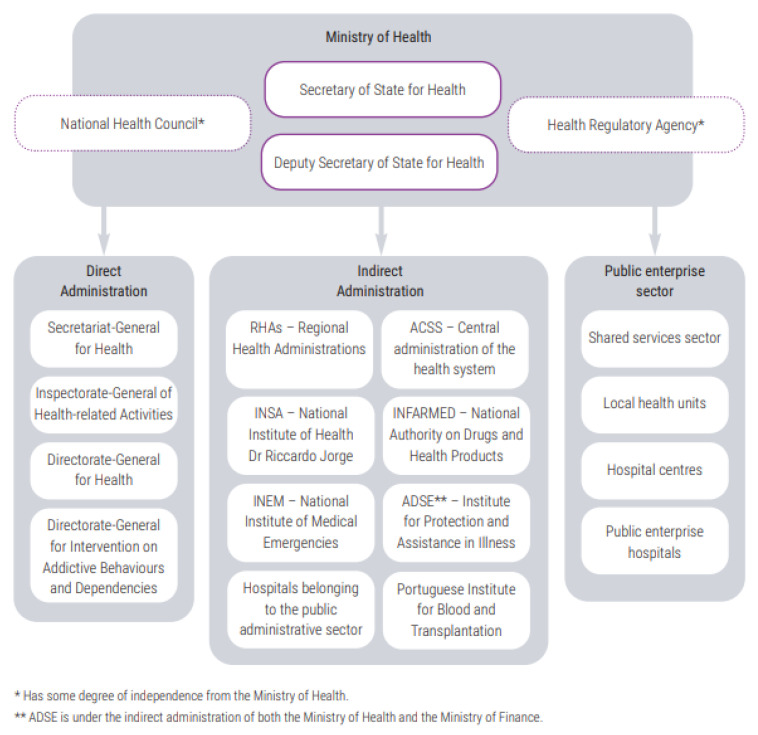
Organizational chart of the Portuguese Ministry of Health. Source—Oliveira et al., 2022 [7].

The hierarchical management structure of the NHS is composed of the Directorate-General for Health (DGH), an entity with responsibilities in health promotion and disease prevention, also playing an important role in the dissemination of healthcare and public health programs; the Central Administration of the Health System (ACSS), which manages financial and human resources and infrastructure; and the Regional Health Administrations (RHAs), which are in charge of the implementation of the national health policy at the regional level, as well as the coordination of all levels of healthcare, namely at regional and local levels [7]. Each RHA comprises Health Center Groups (HCGs), which are responsible for the management of primary healthcare [7,22,25] (Figure 1). It should be noted that, in the past year, the structure of the NHS and the responsibilities assigned to each actor have been debated at the political level. The discussion focuses on the implementation of a new model, separating the political decision (Ministry of Health) from the management overview, now in the hands of a kind of “CEO” of the NHS. This new organic structure also foresees that, in the next couple of years, RHA responsibilities will be integrated with a different regional entity, the Regional Coordination and Development Commissions, which are NUTS II regional public institutions responsible for promoting the coordinated actions of decentralized services at a regional level and for managing community programs based on funds allocated to Portugal by the European Union.

In Portugal, the Ministry of Health centralizes most planning and regulatory activities. Although the five RHAs are responsible for strategic health management at the local level and played a key role in the management of the COVID-19 pandemic, together with the HCGs, the response is still very concentrated in national bodies, which still highlights an overly centralized health system [26]. According to Nunes and Ferreira (2022) [27], such centralization goes along with the growing signs of the inefficiency of the state in the management of health services and the difficulties of citizens in accessing services. The authors mention public–private partnerships, the contracting of services, and co-operating public hospitals as examples that show a lack of state responsibility for health services, which began to be introduced at the beginning of the 21st century. 

Inequality in healthcare access continues to be one of the most relevant points of criticism of the NHS, since it persistently focuses on disease, providing greater resources to central hospitals as opposed to primary healthcare. Moreover, there is little interconnection between health institutions (e.g., public hospitals and primary healthcare; healthcare providers and social providers), difficulties in sharing information, and a lack of funding and incentive in collaborative activities [7]. It was on this premise that, over the last decade, the national health policy has rethought decentralization at the primary healthcare level, making these health units closer to the citizen in an attempt to provide proximity care with higher-quality services, emphasizing global public health issues [27]. 

In 2013, through the framework for the transfer of responsibilities to municipalities (Law no. 75/2013), the Portuguese government reassigned more responsibilities to local authorities within the scope of health and well-being promotion. However, according to Simões et al. (2017) [25], the role of municipalities in this field remained secondary and somewhat limited, given the lack of instruments and resources capable of responding to their new responsibilities (e.g., healthy behaviors and environmental health, among others) [7]. 

The recent administrative decentralization process (Law no. 50/2018 and Decree-Law no. 23/2019) reinforces such efforts, transferring new responsibilities to local governments that are important in the scope of health promotion and disease prevention. In terms of primary healthcare, local governments now have a set of responsibilities involving the management of infrastructures and non-professional human health resources, logistics issues, and the implementation of a Municipal Health Strategy [7]. The legislation also foresees the creation of a Municipal Health Council with local stakeholders, who become responsible for contributing to the design of a health and well-being policy at the local level and issuing a judgment on the Municipal Health Strategy.

The transfer of responsibilities, however, has been regarded with some reluctance by local governments. As of June 2022, only 51 of 308 municipalities had accepted such new responsibilities regarding health [7]. According to Santinha and Perelman (2022) [28] and Julião and Nunes (2019) [24], their reluctance may be caused by four aspects: (i) the commitment of having to guarantee the provision of services adequate to the level provided to date; (ii) the funding transferred by the central government, which, in most cases, may be insufficient to cover expenses; (iii) the organic structure of city councils, meaning that this transfer of responsibilities may imply big changes in their structure; and (iv) the existence of a vision of health and a discourse very focused on healthcare providers, particularly hospitals, so the increase in the responsibilities of municipalities in this field should be regarded with some caution, since their competence is mostly centered on primary healthcare and on the perspective of the prevention and promotion of the population’s health and the determinants associated with it.

## 4. Conceptual Framework Underpinning the Development of Health Strategies at the Local Level

There is a growing recognition in the academic and political arenas that health encompasses multi- and interdisciplinary fields and is influenced by a range of contextual factors, including where people are born, grow up, live, and age, the so-called social determinants of health [29,30]. Dahlgren and Whitehead (1991) [31] published one of the most cited models that explain the impact of these determinants on population health. Based on a stratified structure that encompasses individual lifestyles, social and community networks, working and living conditions, and socioeconomic, cultural, and environmental settings, the authors argued that health policies should be designed from several domains in order to be comprehensive, effective, and efficient.

Based on these assumptions, in 2021, the WHO launched the European Programme of Work, 2020–2025—“United Action for Better Health” (EPW), a new policy that defines four change-promoting initiatives for all Member States to build universal health coverage, to improve the protection of their population from health emergencies, and to build healthy communities, with appropriate public health actions and public policies that ensure a healthy life and well-being for all at all ages. These three interdependent priorities establish the pillars of the WHO’s 13th General Programme of Work, 2019–2023 (GPW 13), are interrelated with the 2030 Agenda for Sustainable Development Goals (SDGs), and are linked to the Triple Billion Targets, goals aimed at improving health and intended to be achieved by 2023.

This means that improving the health and well-being of citizens and communities while reducing health inequalities requires engagement with a range of characteristics rooted in the population and their living environment. These include social, economic, environmental, and geographical planning issues, as well as the wider impacts of policies and decisions across all sectors [5,32]. This is the underlying rationale for the concept of “Health in all Policies”, which emphasizes the need to promote health in the context of where citizens live or work by involving all policy areas and intersectoral networks. Additionally, the concept of “Health for all” highlights the importance of the equitable distribution of resources for health and ensuring that essential health services are accessible to everyone [12,33,34,35,36].

With a key role in linking the central government to society and working closer with communities, municipalities are at the forefront of capitalizing on opportunities to promote citizens’ health and well-being. The local government takes on particular relevance when considering that each location has its own cultural arrangement, which can influence the political, social, and health culture of its stakeholders, thus becoming extremely important in the design of health policies [12]. In addition, the COVID-19 pandemic revealed the importance of municipalities in dealing with such atypical phenomena but with a high impact on citizens’ health and socioeconomics [7]. The Policy Brief released by the United Nations (UN) in July 2020 precisely addressed this issue, presenting a diagnosis of the impact of the pandemic at the community level and mentioning some responses by local authorities considered good practices in this process [37]. What such lessons also show is that decentralization has benefits in a crisis such as the COVID-19 pandemic, particularly if there is a previous tradition of collaboration between different regions and municipalities. On this subject, Shair-Rosenfield (2002) [38] states that although decentralization may be beneficial for everyday governance, emergency and crisis management systems will produce worse outcomes when they fail to facilitate coordination among different levels of government.

Local governments usually have primary responsibilities for planning and delivering services that are crucial to addressing the social determinants of health, including education, transport, housing, and urban planning. They are also often in a strong position to assemble a wide variety of local stakeholders to stimulate action in a way that a single sector by itself cannot [12]. This has led several authors to argue that decentralization enables responsiveness to local needs and values, supports health policymaking that aligns with and empowers various stakeholders, enhances community engagement, and strengthens social capital [39,40]. Moreover, local governments provide essential public health support and are committed to creating conditions for healthier living and to enabling intersectoral action to attain greater equity in health [41]. In this sense, local authorities have in their hands a set of instruments (plans, programs, agendas, and strategies) that seek to tackle existing needs at the local level from a multidisciplinary perspective.

However, despite the theoretical relevance of local governments, there is a lack of information regarding the practical design and conditions for the success of local policies on health promotion and disease prevention. The scoping review conducted by Quiling et al. (2022) [12] shows the existing arbitrariness in the development of health strategies at the local level. These findings led the authors to provide a set of recommendations on this matter, including ensuring citizens’ participation in the design of strategies, developing low-threshold approaches to health and well-being promotion and the systematic integration of local partners, establishing intersectoral measures so that Health in All Policies can succeed, strengthening theory development and research for the continued development of health promotion, and hence, involving stakeholders from academia.

These findings are in line with those of other authors, such as Weber (2019) [42] and Quilling et al. (2020) [12], who argue that context-oriented interventions at the local level have not received the desired attention in practice and research, and therefore, a new consciousness of the importance of health and the recognition of the responsibilities of local governments and sectors beyond the health sector has to be encouraged. In Portugal, as already mentioned, the new legal framework endowed local governments with a more formal mandate to develop a Municipal Health Strategy, but again, no guidance on how to develop the strategy was provided, raising doubts and uncertainty on how to proceed on this matter [43]. 

Nonetheless, the authors share the belief that health strategies at the local level should be driven by an understanding of the main determinants of health, as outlined in Dahlgren and Whitehead’s model. This involves recognizing the various pathways through which decisions can impact the health conditions of citizens and communities, promoting intersectoral collaboration, and adopting a participatory planning approach that encourages the exchange of ideas and consensus building. However, they also note that the success of such strategies is highly dependent on political will and effective decision making.

**Figure 2:**
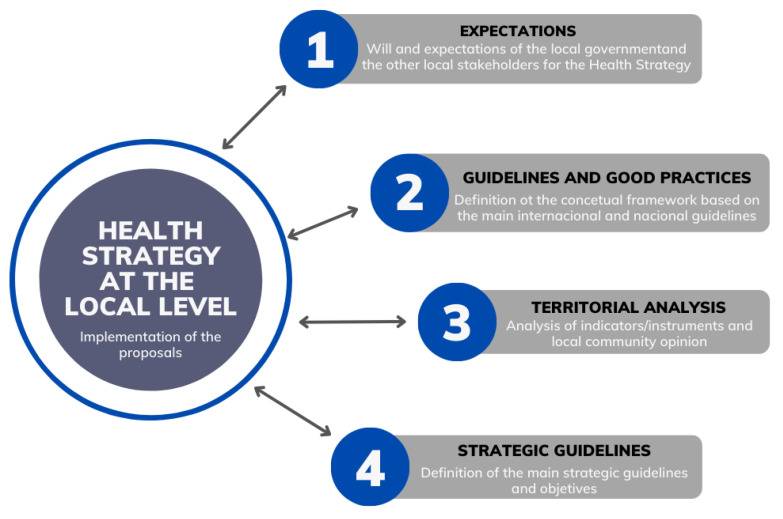
Conceptual framework supporting the development of health strategies at the local level. Source—created by the authors.

Bearing these guidelines in mind, we propose a conceptual framework based on four phases to support the development of a health strategy at the local level (Figure 2). The use of such an approach will allow local governments, together with local stakeholders, to be proactive rather than reactive, to prepare for the future, to help set up a sense of direction, and to contribute to establishing a realistic action plan in line with a vision and mission for the health and well-being of the local community.

The first phase focuses on the vision and mission that a municipality wants to achieve with respect to health and well-being. This “perception of expectations” involves understanding the local government’s reasons regarding the health strategy and interpreting their expectations based on a set of information about the specificities of the territorial context under study. This step aims to establish a consensus-building process with the local government, resulting from a mutual procedure of reflection and conceptualization. It is also important to involve a range of political actors, city council technicians responsible for the relevant areas, and key local stakeholders to ensure that the results are comprehensive and incorporate a diversity of perspectives about the work to be undertaken.

The second phase involves defining and discussing orientation guidelines with the municipality that can frame the topics, potentialities, and barriers associated with the development of a health strategy at the local level. The guidelines should highlight the main societal challenges linked to the health field, creating an opportunity to emphasize themes directly and indirectly related to health and well-being in various international contexts. This will promote a learning process for the local government’s executives, technicians, and other local stakeholders on how to address health and well-being from a non-healthcare provider perspective.

The third phase addresses the territorial context that is under study. This includes collecting and analyzing secondary data, mapping, and listening to the voices of the community and the main local stakeholders. This participatory process must be encouraged to enhance the analysis of the local features and explore potential responses in the health field. This process also allows the building of bonds between citizens and stimulates the debate on health and its influence by all the health determinants, once more creating the conditions for a collective learning process. This participatory process may include several tools, including interviews, focus groups, nominal group techniques, and the application of questionnaires. The choice of tools to use always depends on the participants and the context.

The fourth phase consists of defining the strategic guidelines, the goals, and an action plan. The success of a health strategy largely depends on the level of commitment to those guidelines. On the one hand, political will plays a vital role in influencing individuals and local actors, encompassing Health in All Policies, and providing resources for implementation. This means that policymakers must be willing to participate in the entire process. However, the action plan can only be implemented if all stakeholders are directly involved in designing and implementing the health strategy. In this sense, it is critical to both develop and maintain local capacity building and cooperation while continually sustaining that capacity by creating clear channels for coordination and collaboration and by communicating regularly.

Finally, it is worth mentioning that the local government can play a crucial role throughout this process. For instance, it can demonstrate strong political will and leadership by prioritizing health and committing to the goals and guidelines outlined in the health strategy. Moreover, the local government can integrate health considerations into various sectors and policies, thereby promoting a holistic approach to population health. In addition, establishing mechanisms to monitor and evaluate the efficient use of resources is essential to ensuring their effective utilization. Furthermore, the local government can facilitate the active involvement of stakeholders by creating platforms for collaboration, consultation, and dialogue. Lastly, the government can also engage in effective communication campaigns to raise public awareness about the health strategy and its goals, which will help educate and inform the public about the strategy’s importance and encourage their support. 

**Figure 3:**
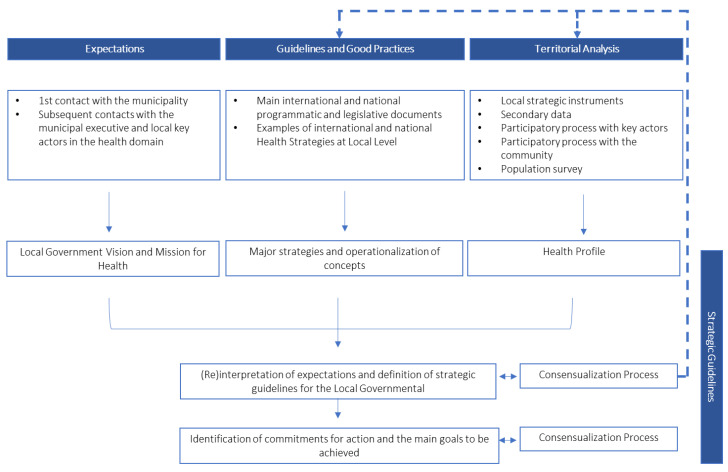
Methodological model. Source—created by the authors.

The following diagram (Figure 3) depicts the methodological model to be adopted in the development of a health strategy at the local level based on the conceptual framework. It consists of the four phases previously mentioned, three of which act or can act complementarily and almost simultaneously, while the fourth phase builds upon the previous ones.

## 5. Case Study: Municipality of Arouca

### 5.1. Brief Characterization of the Municipality

**Figure 4:**
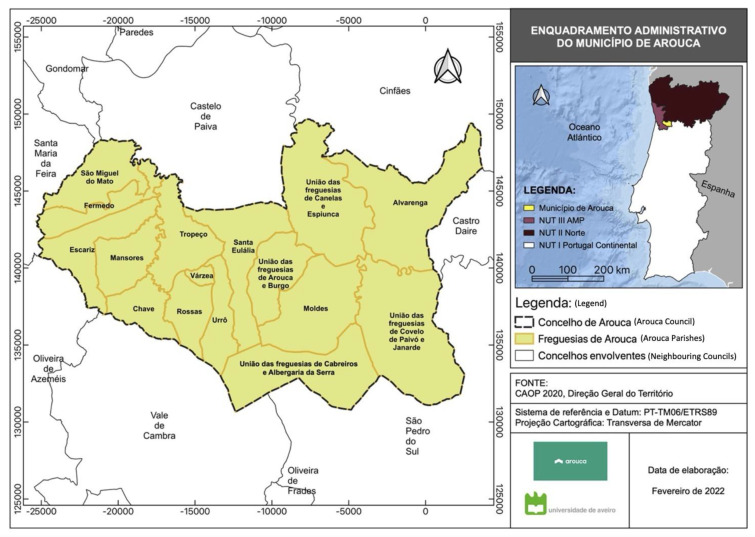
Administrative framework of the municipality of Arouca. Source—created by the University of Aveiro.

The municipality of Arouca is located in the southeast part of the Metropolitan Area of Porto, NUTS II Região Norte of Portugal. This municipality is 329 km^2^, subdivided into 16 parishes, as shown in Figure 4. The municipality’s positioning displays two realities: a western area that is more industrialized, with well-connected road infrastructures but with a dispersed population, and an eastern area that is more mountainous and depressed from the demographic, social, economic, and infrastructural viewpoints. The municipality’s environmental amenities are well known, providing an additional benefit from a touristic perspective.

As of 2021, the municipality of Arouca comprised 21,154 inhabitants, with a population density of 64.3 people per sq. km. Similar to the country’s demographic trend, Arouca has a high aging rate, associated with the greater dependency of the older population. Citizens tend to be less educated in areas more distant from the municipality’s main urban settlement; still, there is an increase in schooling in general, particularly at higher levels. Numerous citizens work in the industrial sector, with the primary sector nonetheless increasing in the last couple of decades. There is also a low unemployment rate compared to the national figure, but still an income far from the reality felt in nearby territories.

Regarding transportation, the municipality does not provide a transport network capable of meeting the needs of the population, who are very dependent on the use of their own vehicle. The fact that the territory is mountainous and dispersed does not facilitate the use of non-motorized means of transportation (soft mobility). In this respect, we can observe that the main services are difficult to access by the most remote population.

**Figure 5:**
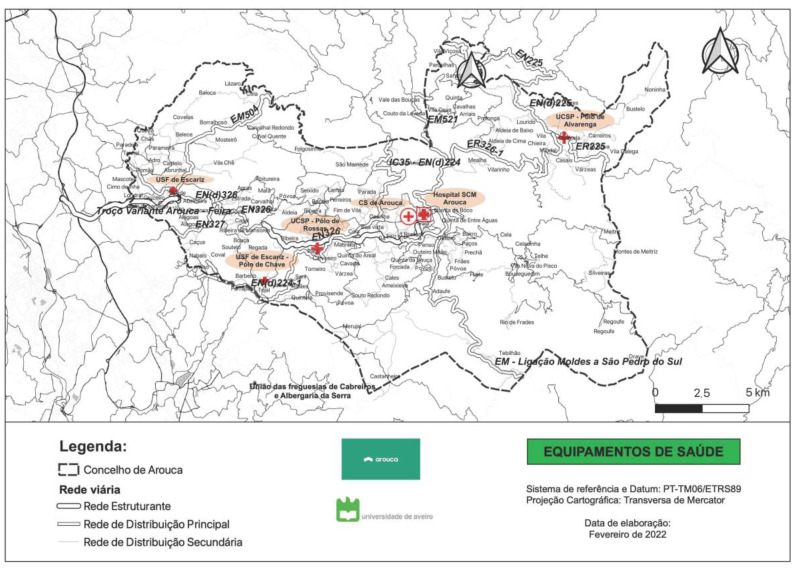
Health facilities and pharmacies. Source—created by the University of Aveiro.

As for healthcare provision, the municipality of Arouca is under the responsibility of ARS Norte (at the regional level) and ACeS Feira/Arouca (at the local level). In the municipality, there are primary healthcare units, with poles distributed throughout the various parishes, and a private hospital under the authority of Santa Casa da Misericórdia (a non-profit-making local entity with a charitable historical background and a strong social role in Portugal) (Figure 5). With respect to access to these services, some remote places can take up to 40 min. The main secondary healthcare providers are also distant from the municipality, taking up to more than 60 min in the easternmost places.

### 5.2. Methodological Approach

The methodological approach taken in Arouca’s Municipality Health Strategy was based on the framework presented in Section 4. Following Figure 3, we now explain how the conceptual framework was applied to the case study.

The first phase encompassed meetings with the municipal executive and key players in the health field in order to understand their expectations regarding the Municipal Health Strategy, as well as their vision and mission for health and well-being in Arouca. This first step was key to understanding how these actors saw the role of local governments regarding health promotion and disease prevention.

The second phase of the project involved developing orientation guidelines, which included analyzing and synthesizing the main international guidelines issued by the World Health Organization, such as Health 2020, Health 2021, and Healthy Cities: effective approach to a rapidly changing world. National guidelines, namely, the National Health Plan, the Regional Health Plan, the Local Health Plan, and other strategies/plans that may impact citizens’ health and well-being, were also analyzed. Furthermore, a set of local health strategies in the national and international contexts was analyzed to provide a frame of reference regarding the topics that a health strategy should include. This second step was crucial in providing the city council with a report that synthesizes the range of topics that local governments can consider in a health strategy.

In the third phase, the territory was analyzed through an exhaustive collection of secondary data: the main local plans and the main initiatives developed in the health and well-being field. At the same time, a participatory approach with the local community, both seniors and young people, was carried out in order to provide a more qualitative portrayal of the populations’ health and well-being. This third step led to the development of Arouca’s Health Profile.

The fourth phase included the definition of the main strategic axes and objectives to outline the main guidelines for community health and well-being in the short and medium/long term. This phase involved consecutive consensus-building processes with the local government and other stakeholders. At the end of the process, an action plan was created.

### 5.3. Operationalization of the Approach

Arouca’s Health Strategy rested on its ability to reflect the social, cultural, well-being, and economic realities of its territory and provide support for the challenges they face. As such, the health strategy strongly relied on the involvement of different entities and the community to successfully design and implement public interventions. However, the operationalization of the strategy came with challenges. The first one was related to the time that the local government was expecting the process to take: one year maximum. Accordingly, the development process of the strategy included four phases.

The first phase lasted two months and included initial meetings with the municipal executive and consultation with local entities, culminating in the delivery of a first report on the “Conceptual Framework”.

The second phase took place over the following two months and consisted of a collection and analysis of secondary data (directly and indirectly related to the populations’ health and well-being), forming the basis of the second report, called “Characterization of the Municipality”.

The next phase involved a five-month period and was concerned with listening to the community throughout different auscultation processes, as well as collecting and analyzing previous and ongoing local initiatives on local health promotion. This third phase concluded with the “County Health Profile”, materialized in the third report.

Finally, the fourth phase, which took place over the following five months, involved the definition of the strategic axes and intervention proposals in a lengthy process of coming to a consensus with the municipality, resulting in the fourth report: “Main Strategies and Action Plan”. At the end of this process, Arouca’s Health Strategy was submitted to the Municipal Health Council and the Municipal Assembly for approval.

The following subsections describe the process in more detail.

#### 5.3.1. Managing Expectations and Defining the Conceptual Model

The process began with a formal meeting with the municipal executive and other stakeholders responsible for implementing the Municipal Health Strategy, including the director of the ACeS Feira/Arouca. The primary objective of this meeting was twofold: first, to provide an overview of the municipality’s significant health challenges, and second, to identify the agents’ perspectives on what a Municipal Health Strategy should entail. Additionally, the meeting aimed to collect crucial indicators that were not publicly available but were in the hands of these key stakeholders, which were essential for developing the municipality’s profile.

In the first phase, several visits to the municipality were conducted to identify and analyze the territory. Later, local institutions involved in health and well-being were consulted, as they are already part of the municipality’s cooperative network dynamics in structures such as the “Social Network”. This consultation process aimed to listen to these local entities and understand their expectations regarding the strategy’s implementation. This participatory process also provided a qualitative portrait of the territory by identifying the primary constraints from social, economic, and health perspectives, the impact of the COVID-19 pandemic on municipal dynamics, and the main areas of intervention.

For this consultation process, a formal presentation was made in the library hall to all participants on the aims of a Municipal Health Strategy and the determinants that influence the population’s health and well-being. The methodology used involved dividing the stakeholders attending the presentation into groups of 12 members, followed by conducting focus group sessions to discuss the issues listed above in an open, free, and participatory manner. At the end, a summary of the main points discussed was presented jointly by all participants in the various groups.

#### 5.3.2. Arouca’s Profile Design Process Using Secondary Data Analysis

During this stage, a vast amount of secondary data were gathered from various areas that can potentially impact the health and well-being of the population, including education, sports, employment, and the environment, among others. The collected data were then classified into eight main areas for characterization purposes:

1. Spatial Context: This area involved an analysis of the geographical location of the municipality and its surrounding areas, including road network and settlement patterns.
2. Demography: The focus of this area was on data related to the resident population, population density, and rates of aging, longevity, and dependency across different age groups.
3. Society, Economy, and Environment: This analysis encompassed the mapping and characterization of educational establishments and indicators related to the educational qualifications of the population. An analysis of the economic activity of the municipality was also conducted, along with an assessment of the average monthly income, purchasing power, and various social support and income-related indicators. Additionally, an evaluation of social vulnerability across different territorial profiles was carried out. Environmental data were collected, including safety data in areas such as water, sanitation, waste, energy, and climate.
4. Social Responses: This area described the various types of social equipment available in the municipality and their respective geographic locations, as well as the population that uses them.
5. Sports and Leisure: This topic focused on the various sports equipment and parks and outdoor leisure spaces in the municipality, as well as their mapping.
6. Mobility: This area involved an analysis of the type of transportation used by the population, including the mapping of urban transport networks.
7. Health and Welfare: This topic gathered several indicators related to birth rate, mortality, and morbidity.
8. Healthcare: This area involved a portrayal of primary healthcare, hospital care, and integrated continued care. The analysis included mapping the municipality’s health facilities and their geographic accessibility, as well as the main reference hospitals and the travel time required to reach them. An assessment of the main health problems affecting the population and the indicators related to the use of hospital care in terms of emergency episodes, inpatients, operating rooms, and outpatient consultations was also conducted.

#### 5.3.3. Arouca’s Profile Design Process following a Participatory Approach: Going beyond Data Statistics

This phase of the study aimed to overcome the lack of statistical information and to obtain qualitative data that closely reflect the reality of the community by consulting with its citizens. The involvement of the community in this process is crucial to enhancing the accuracy of the profile and exploring potential solutions to address the identified issues. Citizen participation enables us to enhance the quality of decision-making processes, improve the context in which we live, and foster connections among community members.

To collect data, focus group sessions and an online questionnaire were applied, with a total of 189 citizens participating, including 73 in focus group sessions and 116 in the group completing the online questionnaire.

The methodology employed in the participatory process involved presenting the work program, outlining the session plan and participation rules, and using an icebreaker to introduce each participant through a popular saying/proverb related to health and well-being. Participants were then asked to use a lotus flower table to describe constraints and potentialities in the municipality related to various health determinants, such as food, social and cultural habits, mental health, health literacy, housing, support for the elderly, health services, communication, and collaboration between institutions and actors. In the second round, participants were asked to suggest interventions that would address the identified problems. Finally, group representatives shared the summary of the results of their respective worktables.

The survey aimed to identify the main constraints in the municipality in the fields of health, social, and economic areas, to identify areas where interventions were necessary to improve the quality of life of the population, and to obtain suggestions for the Municipal Health Strategy. The survey was widely distributed to reach as many people as possible.

The consultation process also involved engaging with students in grades 9–12, with a total of 36 participants. Engaging youth in participatory and educational processes is crucial to promoting health and expanding opportunities for debate and decision making, leading to increased social participation and empowerment. Effective health promotion in schools requires the active participation of adolescents and young adults, as their involvement in planning and managing health strategies can lead to changes in the design and implementation of health programs and policies. Mechanisms must be put in place to ensure their participation and to reflect their needs in policymaking.

The same methodology used in the community focus group sessions was adopted in the round-table discussions with students. Students were divided into groups and introduced themselves using a health-related meme to share their expectations for the activity. They identified constraints, potentialities, and good practices in the municipality, with a focus on territorializing the information when possible. Lastly, each group proposed an idea for an experimental action based on the information gathered, with the aim of collecting contributions for future actions in the municipality.

The information gathered from the focus group sessions was consistently analyzed using the lotus flower technique to identify key areas of discussion based on secondary data. The analysis included identifying the main constraints, potentialities, and intervention proposals for each area, with priority given to those mentioned most frequently by the participants.

Finally, local institutions were asked to provide information on existing health and well-being initiatives in the municipality to help identify areas in need of more intervention. This information was used to develop Arouca’s Health Profile, which included an analytical aspect, consisting of an analysis of the main intervention needs, and a more interventional aspect, based on understanding the existing local main instruments (plans, strategies, initiatives), collecting context indicators, engaging in a dialogue with the main local stakeholders and the community in general, and traveling through all municipal parishes in order to observe the inherent characteristics of the territory.

#### 5.3.4. Reaching the Final Stage: Main Strategies and Action Plan

After the development of Arouca’s Health Profile, the main strategies and intervention proposals were devised to cover a comprehensive perspective on health, encompassing the “Health in All Policies” and “Health for All” concepts. These strategies were designed in accordance with the principal issues outlined in the health profile and the strategic guidelines established by the World Health Organization (WHO) and the General Directorate of Health (DGS), and they were also in line with the National Health Plan, the Northern Regional Health Plan, and the Feira/Arouca Local Health Plan. Once the main strategies were established, a process of consensus building with the city council executive members commenced, resulting, in the end, in five main strategies, namely, (i) enhancing health literacy, citizenship, and communication; (ii) promoting environmental health and improving quality of life; (iii) ensuring safe and inclusive living conditions; (iv) fostering social and territorial cohesion in accessing healthcare; (v) establishing a sustainable institutional landscape.

Upon consolidation of the outlined strategies with the municipality, an action plan was developed to be implemented in subsequent years, comprising structuring proposals that would continue over time, as well as specific proposals to be implemented within a particular period of the year. It is noteworthy that the proposed initiatives were intended to be as pervasive as possible across all outlined strategies and were few in number, given that a Municipal Health Strategy should focus on the most critical points. In this sense, ten actions have been designed to address the overarching key objectives of the five main strategies. A standard form was prepared for each proposal, containing a description, a justification based on the health profile, and a summary of the operational objectives. For each proposal, a standard form was prepared, which includes a description, a justification based on the health profile, and a summary of the operational objectives. The form also specifies the responsibility for implementation, the entities involved, and the alignment with local strategic and operational orientations. Additionally, monitoring and expected goals, along with monitoring indicators, are included in the form. The inclusion of a monitoring process in each proposal enables the evaluation of the success of each action. This evaluation provides insights in the medium to long term, facilitating the identification of possible improvements to the plan and the reorganization of priorities.

**Figure 6:**
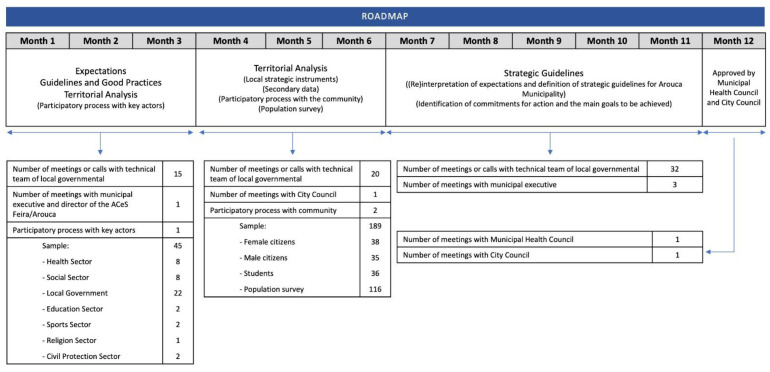
Line of action of the Municipal Health Strategy. Source—created by the University of Aveiro.

Figure 6 illustrates a timeline of the actions undertaken during the development of Arouca’s Municipal Health Strategy based on the previously detailed phases. Each stage includes information on the number of interactions and participants involved in the strategy’s elaboration. It is important to note that the strategy consists of four comprehensive reports, namely, (i) background and theoretical framework; (ii) health determinants: secondary data analysis; (iii) population health profile; (iv) main strategies and intervention plan.

## 6. Discussion

The aim of this article was to propose and discuss a conceptual and methodological approach to define and implement a health strategy as an attempt to change, through practice, the dominant culture regarding the design of health policies at the local level. In particular, concerning the recent decentralization process in Portugal, we propose a set of key steps for local governments to consider in the development of such strategies. International organizations, such as the World Health Organization [44,45] and the European Commission (2007) [46], emphasize the proximity of local governments to communities, mainly due to their crucial role in health and well-being promotion, disease prevention, and fostering healthier territories for and with citizens.

For this to take place, designing a health strategy requires, from the outset, political will and collaboration among various actors and the community, with local governments assuming responsibility for dynamizing the whole process and guaranteeing resources for its implementation [47]. It also requires an understanding of the reasons supporting the development of the health strategy. In other words, what are the local government and key actors’ expectations regarding the development of a health strategy at the local level?

The implementation of the first step in our case study, Arouca, proved to be a crucial point. The political discourse mainly focused on curative health care, i.e., human resources and health facilities, rather than health promotion and disease prevention, which are areas where local governments can make a difference. As argued by Aadahl et al. (2023) [48] and Baum and Friel (2017) [49], governments find it challenging to analyze and implement policies based on health promotion and the various conditions that influence it, such as urban planning, education, and social aspects, as they do not see these areas as directly related to health. The first major challenge was to adopt a pedagogical approach to change the local government’s mindset, i.e., to demonstrate that the municipality’s competence should focus on health promotion and disease prevention, leaving the curative model to the Ministry of Health entities and healthcare providers. The social determinants of health conditions and the concepts of “Health in all Policies” and “Health for All” proved to be useful in the debates held with Arouca’s City Council executive members and technicians. A similar reaction was found among the other stakeholders, as their mindset was centered on curative care as well. In this case, however, the help of the local government was paramount in starting a pedagogical dialogue to show how, conceptually, all stakeholders could look at and contribute to improving the population’s health and well-being conditions.

This entire pedagogical process and change in mentality were only possible due to the development of orientation guidelines that brought together the main international (e.g., Health 2020; Health 2021; Healthy Cities: effective approach to a rapidly changing world) and national guidelines (e.g., National Health Plan; Regional Health Plan; Local Health Plan; other plans/instruments that relate directly and indirectly to the health domain). Similar strategies already implemented in some European countries (e.g., Spain, Australia, the United Kingdom, France, and the United States of America) were also collected. All this information, included in the second phase of our proposed conceptual model, provided a solid basis for how to structure and implement a health strategy at the local level.

The following step of our model encompasses the analysis of the territory, which includes, on the one hand, the collection and analysis of secondary data and, on the other hand, a community consultation. On the premise of the health determinants model, a broad spectrum of indicators has to be analyzed. In the case of Arouca, however, obtaining some data was significantly difficult, particularly at the local level and especially in the area of health and healthcare. Most of these data are under the jurisdiction of the Ministry of Health, which, due to its bureaucratization and data protection policy, made access to them hard. On the other hand, some of the available data were outdated and aggregated to regional or national contexts. Despite technological advances in tools that allow data collection and improve data analysis and visualization, this difficulty is reported by several other authors, as the use of such data remains limited in many settings, especially at lower levels such as districts or communities [50].

At this point, a data mismatch was identified, specifically concerning the initiatives implemented at the local level. The data provided by the municipality and other stakeholders were insufficient compared to what was being executed. During the consensus-building process of the health profile and the development of the main strategies, this discrepancy was discussed, highlighting the need for another round of dialogue with city council technicians and local stakeholders. This experience taught us the importance of cross-checking data from various sources and proactively questioning their accuracy.

In order to partly fill this gap, our methodological model focuses on a strong participatory process involving the community, complementing existing statistical data. This premise is supported by the WHO (2012) [41] and Saltman and Bankauskaite (2007) [51], who state that gathering citizens and key actors around a table to discuss effective and efficient local policies is one way to collect and characterize the territory and its community [51]. In the case of Arouca, a participatory process involving the community, including both young and old people, was carried out to discuss constraints, potentialities, and good practice examples in the area of health and well-being. As indicated in the conceptual framework, listening to these stakeholders is essential to discuss the determinants of health, raise awareness about behavioral change, and make them active agents in the design of local policies [52]. The OECD has also collected evidence and data supporting the idea that citizen participation in public decision making can generate better policies, strengthen democracy, and build trust [6].

However, when analyzing citizen participation in local strategic planning processes, frequent hurdles can be found. Participation may be limited to the moments provided for in the legislation, which are clearly insufficient, or the population may be overwhelmed with numerous participatory processes that overlap, most of the time without producing visible results, causing citizens to mistrust this type of process [53,54]. According to Antonini (2015) [55] and Delgado (2013) [56], the motivation of individuals to participate in consultation processes must be grounded in a sense of credibility, where they see that their opinion will have value and an impact on their personal and collective lives.

In our case study, some difficulties arose in motivating the population to participate in focus group sessions during the consultation process, precisely because of the mistrust that what was discussed would never be reflected in practice, leaving the documents “closed in a drawer” and, as such, never going into practice. Our strategy was to encourage citizen participation through the strong involvement of the parish mayors, who were tasked with ensuring the presence of citizens from their parish while guaranteeing criteria based on age and gender. Still, some parishes were not fully involved, thus limiting the participation of some citizens. Efforts were also made to change the mindset of the population through an interactive methodology that was accessible to all participants, similar to what was implemented with members of the municipality and local stakeholders.

It is crucial to highlight the active participation of citizens during the participatory process, as well as the effectiveness of parish mayors in facilitating and mobilizing citizens. The parish mayors were readily available whenever needed, allowing for a better grasp of the local social, economic, environmental, and spatial challenges, opportunities, and requirements.

Overall, the health strategy aimed to have a cross-cutting character, ensuring a broad vision of health and well-being for the municipality. Citizen engagement served to change the way that health was considered and was a starting point for the inclusion of citizens in the decision-making process. The younger community was also given a voice to debate the issue and express their short-term expectations. Students were invited to outline pilot projects that they would like to see implemented in the municipality with regard to health and well-being.

This third phase of the project concluded with the development and delivery of Arouca’s Health Profile. However, once again, a discrepancy arose between the local government’s perception and that of the community, indicating a lack of collaboration and communication among the government, stakeholders, and the community. According to Delgado (2013) [56], transparency and credibility in the results obtained are fundamental for a greater involvement of the population. Regarding communication and interaction between the local government and the community, namely in the discrepancy between reality and perception, Kim and Kreps (2020) [57] state that ineffective communication between government agencies and citizens can generate confusion and misunderstandings, and there should be clear communication adapted to the different target audiences [58].

The fourth phase of our conceptual model focuses on defining the main strategic axes, operational objectives, and action plan. A crucial aspect is to involve local government executives and technicians in a consensus-building process, which creates the necessary conditions to combine scientific expert knowledge with the practical knowledge existing in local communities, the voices of citizens, and the receptivity and responsiveness of governments through institutional change [59]. Thus, the conditions for dialogue must be clearly defined, ensuring that strategies with greater social acceptance are included. Ultimately, a health strategy at the local level should result from a collaborative process that involves a diverse set of people with different perspectives and abilities to implement an action plan, rather than only a vision of one party producing something for the community.

Our case study showed that the process of designing the main strategies and the action plan is complex, time-consuming, and not without discussions, particularly with the executive members of the local government. The differences observed between the community’s perception of the local health and well-being needs and the local government’s perception contributed greatly to the intricacy of the process. In the view of the executive of the city council, the problem mainly lay in the absence of communication between the local government and the community, so the problem essentially arose from the perspective, probably distorted by the citizens, of what was being implemented in the municipality of Arouca. After a new period of analysis of the health profile, an understanding was reached on the main strategic lines to be adopted by the local government, in conjunction with various stakeholders, in order to socially legitimize the local strategy action plan. In addition, one of the essential points lay precisely in improving communication channels between the local government, stakeholders, and the community.

As a final remark, three points are worth noticing. The first is that, according to our conceptual framework model, the development of a health strategy at the local level requires the involvement of a multidisciplinary team. For our case study, the multidisciplinary team included experts from various areas (e.g., sociology, psychology, health, health technologies, gerontology, spatial planning, geographic information systems) to enrich the scientific and technical components of the health strategy. There was also a concern about adopting technical language in order to make the reports accessible to political actors and the community. Experts in civic participation were also involved during the consultation process to ensure that the strategy was inclusive.

The second point relates to the main challenges expected in the short run in implementing a health strategy at the local level. Looking at Arouca’s process, the first challenge concerns the ability to transmit the political power’s message to the community and how this information can be articulated with other local institutions. On the other hand, it is important to identify a group of individuals in each community who are responsible for influencing a change in the group’s behavior. We call these individuals influencers or enthusiasts in that they can help the local government in transmitting a message that ensures the promotion of health and well-being, as well as participation in the outlined initiatives. In this regard, the Municipal Health Council itself plays an important role in how it can develop a communication strategy and implement the initiatives.

The third and final point is to emphasize once again that our conceptual framework was developed to involve various local stakeholders, including scientific, political, and technical perspectives, with constant interaction throughout the process. As shown in Figure 6, there was strong interaction, sometimes daily, between the scientific team, the city council, and other local stakeholders. The use of this framework also created favorable political conditions by inviting opposition parties to participate in the process from the outset, leading to positive feedback.

## 7. Conclusions

The governance of health systems has undergone significant changes in recent years in pursuit of health as an integral part of well-being through both whole-of-government and whole-of-society approaches. This political will has been reinforced by various international organizations, such as the World Health Organization [38]. The idea is to promote joint actions by health and non-health sectors, public and private stakeholders, and citizens for a common interest and achieve a consensus supported by structures and mechanisms that facilitate collaboration. In this context, decentralization processes, aimed at making local governments more autonomous and enhancing their decision-making capacity, have gained particular interest.

Recent legislative and political decisions in Portugal have emphasized the crucial role of local governments in leading new approaches to governance for health. However, there is still some skepticism about differentiated, non-traditional approaches based on new multistakeholder governance arrangements, with an increased role played by local governments in the health and well-being field. Moreover, within the current framework of decentralization, there are no guidelines or indications on how to structure and plan a health strategy at the local level.

The absence of guidelines resulted in the development of a conceptual framework that can be replicated in various contexts and at different levels of governance. In this sense, the framework is being applied in other contexts and has proven to be equally successful and adaptable to their unique characteristics. The framework’s approach to gathering data from diverse sources and utilizing various participatory methodologies enhances the conclusions and the definition of the strategy, resulting in a reliable representation of each community’s health and well-being.

It is worth noting, as a final remark, that this approach raised new questions and ways of thinking about local problems and needs, turning citizens into agents of change in planning for the present and future. Nevertheless, there is still significant room for improvement in all areas related to health and well-being to encourage professionals and the population in general to adopt new attitudes toward health promotion and disease prevention. Additionally, sustaining this institutional capacity for upcoming strategies requires continuing to adopt new multistakeholder governance arrangements with a greater capacity for communication and collaboration. However, uncertainties still prevail in this regard: changing the way that the local government and the community work together, forging strategic partnerships, and opening communication channels are still in the very early stages.

## Data Availability

Not applicable.
